# Ultraviolet Light-degradation Behavior and Antibacterial Activity of Polypropylene/ZnO Nanoparticles Fibers

**DOI:** 10.3390/polym11111841

**Published:** 2019-11-08

**Authors:** Guangyu Zhang, Yao Xiao, Jiawei Yan, Ningwei Xie, Rong Liu, Yu Zhang

**Affiliations:** 1 National & Local Joint Engineering Research Center of Technical Fiber Composites for Safety and Health, School of Textile and Clothing, Nantong University, Nantong 226019, China; zgyu85@ntu.edu.cn (G.Z.); xyt123asd@163.com (Y.X.); ningweixie@163.com (N.X.); z.yu@ntu.edu.cn (Y.Z.); 2 Faculty of Textile Science and Technology, Shinshu University, 3-15-1, Tokida, Ueda, Nagano 386-8567, Japan; 19fs110c@shinshu-u.ac.jp

**Keywords:** ZnO nanoparticles, polypropylene fiber, ultraviolet light-degradation, antibacterial

## Abstract

Herein, ZnO nanoparticles (NPs) were synthesized using zinc acetate and an amino hyperbranched polymer. The methods of transmission electron microscope (TEM) and X-ray Diffraction (XRD) were applied to the characterization of ZnO NPs. Polypropylene (PP)/ZnO fiber was prepared using 1–5 wt% ZnO NPs via melt spinning. The ultraviolet light (UV)-degradation behavior, antibacterial activity and mechanical properties of PP fibers were characterized. The PP fiber filled with ZnO NPs presents better mechanical properties and the resistance to UV light degradation. For the best effect, the contents of ZnO NPs were set 4 wt% in PP fiber. FTIR analysis shows significant photo-degradation of PP induced by UV irradiation and a remarkable reduction in the UV degradation of the fiber filled with ZnO NPs. It is also observed that the ZnO NPs-filled fiber has good antibacterial actives against *Escherichia coli* and *staphylococcus aureus.*

## 1. Introduction

Polypropylene (PP) fiber has been extensively applied in the textile industry because of its low cost, high strength, and resistance to chemicals. However, the physical and mechanical properties of PP materials are much susceptible to photo-irradiation. The photo-degradation of PP macromolecules usually results in decreased service life, limiting the application of PP fibers [1,2,3]. To increase the resistance of PP fibers to ultraviolet rays and the photo-oxidative degradation, organic and inorganic particles were used as fillers for anti-UV applications. Nevertheless, organic UV absorbers are not stable especially at the high temperature and pressure [4,5,6]. Inorganic materials able to withstand harsh process conditions such as TiO2, ZnO, MgO, MMT, and CaO have attracted wide attention. Among them, ZnO nanoparticles (NPs) have been researched a lot because of their low price, unique physical properties brought by the size effect, and potential applications in UV blocking materials [7,8,9,10,11,12].

As reported, ZnO as a filler can not only enhance physical and mechanical properties, but also improve the resistance of PP materials to ultraviolet rays and microorganisms [13,14,15]. More recently, the antimicrobial properties of ZnO NPs have been discovered. Compared with silver NPs, ZnO NPs are expected to provide more affordable and safe solutions in the future [13,16,17,18]. Zhao et al. studied the photodegradation characteristics of PP materials filled with ZnO NPs. The evolution of the carbonyl absorption band at 1780 cm^−1^ obtained by FTIR analysis showed that UV irradiation was more likely to cause obvious photodegradation of PP [1]. Karami et al. [14] investigated the impacts of UV-irradiation exposure on the structure and properties of ZnO NPs-filled PP fibers. Both carbonyl and hydroperoxide indexes were found increasing due to the growing UV-irradiation exposure time; moreover, PP nanocomposite fibers achieved a smaller increase in these indexes than unfilled PP fibers. Yoann et al. [19] used ZnO NPs as a fungicide to synthesize antibacterial PP surfaces. The ZnO-functionalized substrate exhibited an antibacterial response in *Escherichia coli (E. coli)* and the antibacterial activity of ZnO-PP was significantly higher than pure PP.

Notably, ZnO NPs with large specific surface area and high surface energy are difficult to disperse homogenously, leading to agglomeration [20,21,22]. In our previous study, an amino hyperbranched polymer (HSDA) was synthesized and used to control the synthesis of ZnO NPs. It also served as a binder to impart and fix the NPs on cellulose fabric, thereby presenting antimicrobial properties [23,24]. This research work is mainly aimed to synthesize high dispersion ZnO NPs, to develop PP/ZnO nanocomposite fibers by melt spinning, and to investigate UV light-degradation stability, antibacterial and mechanical properties of the prepared fibers.

## 2. Materials and Methods

### 2.1. Materials

Polypropylene powder (F401 melting temperature 163 °C) used was from Yangzi Petrochemical (Yangzhou, China). Zinc acetate, lithium hydrate, and ethyl alcohol (95%) were purchased from Guoyao Chemical Technology Co., Ltd. Shanghai, China. The hyperbranched polymers (HBP) were prepared as described in our previous paper [23].

### 2.2. Preparation of ZnO NPs

Zinc acetate (1.83 g) was completely dissolved in 100 mL of the ethanol solution (95%), refluxed at 80 °C in the flask. After the HBP with a concentration of 2 g/L was added, the mixture was stirred in a magnetic stirrer for 3 h. Then the lithium hydroxide ethanol solution (50mL, 0.1 mol/L) was added dropwise into the solution, stirred for 2 h until the transparency aqueous solution turned into milky white. Finally, ZnO precipitate was produced using a centrifuge at 12,000 rpm for 10 min.

### 2.3. Preparation of PP/ZnO NPs Fiber

ZnO NPs and PP granules were dried at 90 °C for 8 h before the PP/ZnO NPs were melt compounded using a twin-screw extruder (KS220 L/D = 26, Ruiming Co., Ltd., Wuhan, China) at a temperature of 160 °C and a screw rate of 100 rpm. The fiber passed over the godets at a constant draw ratio (λ = 4). The concentrations of ZnO NPs in PP granules were 1%–5%, respectively. A sample of PP was prepared under the same conditions without adding NPs for reference.

### 2.4. Characterization of ZnO NPs

The morphology and lattice characteristics of ZnO NPs were characterized by transmission electron microscopy (TEM) (2010, JEOL, Tokyo, Japan). The crystalline phases of ZnO NPs were analyzed by X-ray diffraction (XRD) (Philips, Amsterdam, The Netherlands) via a CuKα X-ray source at a voltage and current of 40 kV and 30 mA respectively. The UV absorption properties of ZnO NPs were determined by ultraviolet-visible (UV-vis) spectroscopy (UV-3010 Hitachi, Tokyo, Japan).

### 2.5. Characterization of ZnO NPs-Filled PP Fibers

To characterize the photo-degradation behavior, PP and ZnO NPs-filled fibers were exposed to 300 W ultraviolet light (ULTRA-VITALUX, Munich, Germany) for 72 h, 96 h and 120 h, 35 cm away from the light source. The surface morphology of fibers was characterized by scanning electron microscopy (SEM) (6510, JEOL, Tokyo, Japan) and energy dispersive spectroscopy (EDS) (Carl Zeiss, EVO 15, Oberkochen, Germany). The tensile properties of pp and PP/ZnO fibers were studied by Instron 5969 (Boston, Massachusetts, USA) following the ASTM D3822 standard. The sample with a length of 20 mm was stretching between the clips of the tensile tester with a rate of 20 mm/min until the fiber was ruptured. To characterize the UV-oxidative degradation of PP and PP/ZnO fibers, FT-IR (Nicolet, Thermo Scientific, Madison, WI, USA) method was used to measure the carbonyl group on fiber. The spectra were recorded in the range from 400 to 4000 cm^−1^.

DSC (Q-50, TA Instruments, Newcastle, DE, USA) measurement was performed as follows. The PP and ZnO/PP fiber (<10 mg) was chopped and placed in a crucible. Under nitrogen protection, the temperature was firstly reduced from normal state to −20 °C at a rate of 10 K/min and then heated to 200 °C. After 5 min, the same cooling rate was maintained. The temperature was dropped to −20 °C to eliminate heat history. Then it was again raised from −20 °C to 400 °C at 10 K/min, and the secondary heating curve of the fiber was measured. The percentage of crystalline fraction was calculated from these enthalpies as:

(1) $$x=\frac{\Delta H_{f}}{(1-\varphi_{p})\times \Delta H{{}^{\circ}}_{f}}$$

where φ_p_ is the weight ration of ZnO NPs in PP fibers and ΔH^o^f is the reference melting enthalpy of PP (207 J g^−1^).

The antimicrobial activity of ZnO NPs-filled fibers was tested against *E. coli* and *S. aureus* using a shaking flask method following GB/T20944.3-2008 (China) [24].

## 3. Results

### 3.1. Characterization of ZnO NPs

**Scheme 1:**
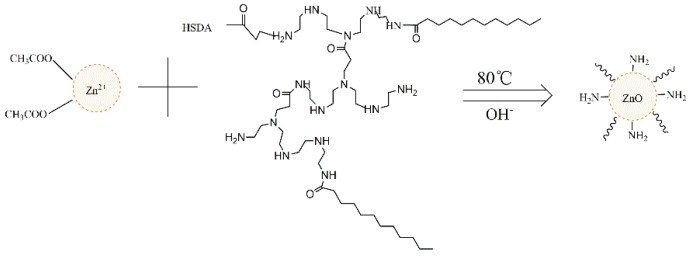
Schematic illustration of synthesis of amino-capped ZnO NPs.

The principle of synthesizing amino-capped ZnO NPs is illustrated in Scheme 1. The solution of hyperbranched polymers was added into the Zn(Ac)_2_ ethanol solution in alkaline medium followed by the hydrothermal reaction. Under alkaline conditions, Zn^2+^ can be converted to Zn(OH)_2_ colloid. In the hydrothermal process, Zn(OH)_2_ colloid dissolves to form Zn(OH)_4_^2−^ and ZnO spontaneously nucleates from Zn(OH)_4_^2−^ solution to form multinuclear aggregates. HBP, with a special structure of internally sealed polymer nanocage, will impede the growth of ZnO NPs and protect them from agglomeration [25,26]. In this way, the size of ZnO NPs can be controlled and the prepared ZnO NPs/PNP hybrid materials obtain good stability in solution.

**Figure 1:**
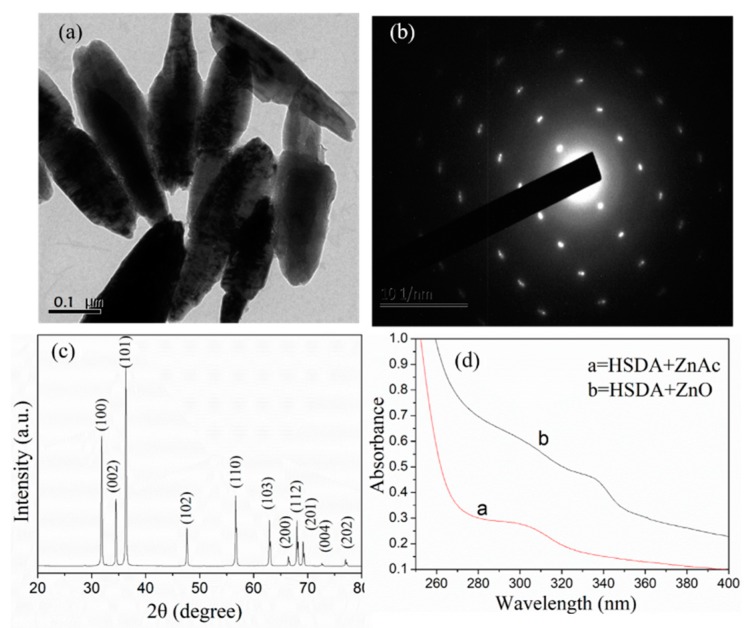
(**a**) TEM images; (**b**) selected area electron diffraction (SAED); (**c**) XRD and (**d**) UV-vis spectra of ZnO NPs.

TEM, SAED, XRD, and UV-vis were used to evaluated the ZnO NPs property. Figure 1a shows the TEM image of the sample, from which numerous NPs with irregular rod morphology can be clearly distinguished. The diameter of amino caped ZnO NPs is about 100 nm. A series of diffraction rings appear in a pattern of the selected area electron diffraction (SAED), as presented in Figure 1b, indicating the crystalline phase of amino-capped ZnO NPs. The XRD pattern confirms the wurtzite structure of ZnO NPs. As shown in Figure 1c, all the peaks of (100), (002), (102), (110), (103) and (112) can be indexed to the planes of wurtzite ZnO and in good agreement with JCPDS data card (36–1451) [27,28]. Interestingly, there is no additional peak corresponding to other impurities in the pattern, implying the obtained ZnO NPs are purely crystalline. The UV absorption properties of ZnO NPs were investigated by UV-Vis spectroscopy, with results in Figure 1d. Compared with the HBP solution, there is a new absorption peak at 340 nm and the prepared ZnO NPs exhibit a strong absorption between 200–400 nm, demonstrating the generation of ZnO NPs [23,29].

### 3.2. Preparation and Mechanical Properties of PP/ZnO Composites:

**Scheme 2:**
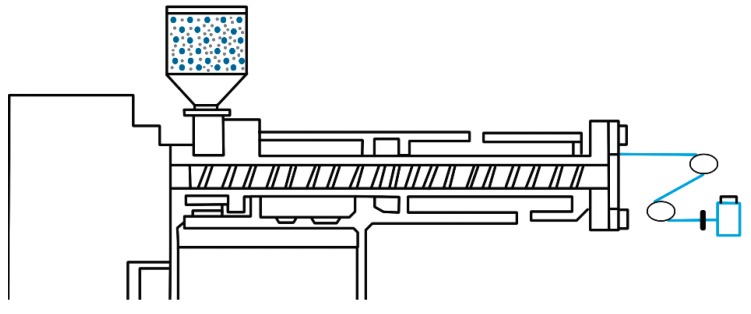
Schematic illustration of synthesis of ZnO NPs-filled fiber.

**Figure 2:**
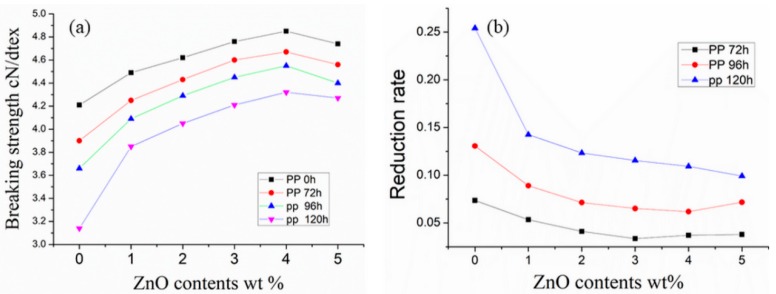
(**a**) Breaking strength of polypropylene (PP) and ZnO NPs-filled PP with different UV-light exposure time (**b**) Breaking strength rate of PP fiber with different UV-light exposure time.

The principle of preparing the ZnO NPs-filled PP fiber is described in Scheme 2. The dispersion of inorganic NPs filled in thermos polymers is much hard, as the inorganic NPs are more likely to be agglomerated [10,30]. To overcome this problem, we synthesized high dispersion ZnO NPs and used it for the modified pp fiber. To verify the strength and UV-degradation behavior of ZnO NPs-filled PP fibers, a test was performed on the tensile strength of PP fiber and ZnO NPS-filled PP fiber exposed to UV-light, as shown in Figure 2. The results indicate that the strength of PP fiber increases from 4.2 cN/dtex to 4.8 cN/dtex with the rising content of ZnO NPs in PP fiber from 1% to 4%. As the content grows to 5%, the strength of PP fiber experiences slighly decreases, since ZnO NPs enhance the toughening effect on PP composite fibers. The nano zinc oxide particles have a small size and a large specific surface area, and a combination of physical and chemical polymer chains is generated when the nanoparticles are blending with the polymer. Thereby, the adhesion between the particles and the polypropylene can be further improved. When the added mass fraction of nanoparticles is large, the range of the flexible interface layer inside the polymer fiber gets increased; when subject to an external force, the internal interface layer will be destroyed before the substrate is damaged, which in turn leads to a decrease in the fiber strength [10,13].

Owing to a large number of unstable tertiary carbon atoms in the PP molecule, the tensile strength of PP fibers was reduced after being exposed to the UV-light. To examine the UV-light degradation properties of PP fibers, PP and ZnO NPs-filled PP fibers were exposed to UV-light irradiation from 72 h–120 h. The results show that as the content of ZnO NPs in PP fiber increases from 0 to 5%, the fiber strength is declined from 25% to 10% under 120 h of UV-light irradiation. With the UV-light and oxygen, the energy can be used to put the tertiary carbon atom; after the removal of hydrogen, the tertiary carbon radicals become the starting active center, thus breaking the macromolecular chain. When the ZnO NPs-filled fiber is irradiated by UV light, the nano zinc oxide shields against part of the UV light, improving the resistance of the ZnO NPs-filled fiber to the UV light.

### 3.3. SEM Images of PP and ZnO NPs-Filled PP Fibers

**Figure 3:**
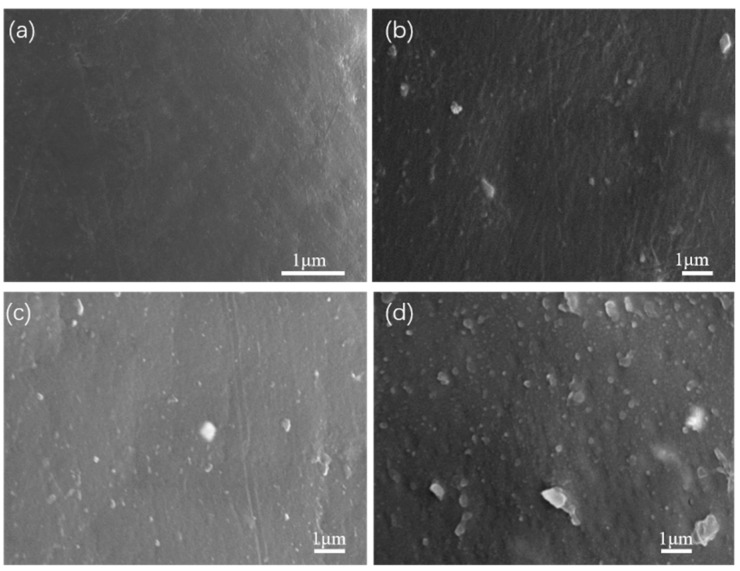
SEM images of (**a**) PP and ZnO NPs-filled PP fibers with concentration of 2% (**b**) 3% (**c**) 14% (**d**).

The surface of PP and ZnO NPs fibers were examined by SEM and EDS to determine the distribution of ZnO NPs on PP fibers. PP and ZnO NPs-filled fibers with different contents are shown in Figure 3. The smooth surface of PP fiber can be clearly found in Figure 3a. SEM images of ZnO NPs-filled PP fibers, as shown in Figure 3b–d, change with the distribution of ZnO NPs. Some aggregations are observed in the fibers filled with 4% of ZnO NPs, as illustrated in Figure 3d. It indicates partial aggregation of ZnO NPs during the preparation of ZnO NPs-filled fibers.

**Figure 4:**
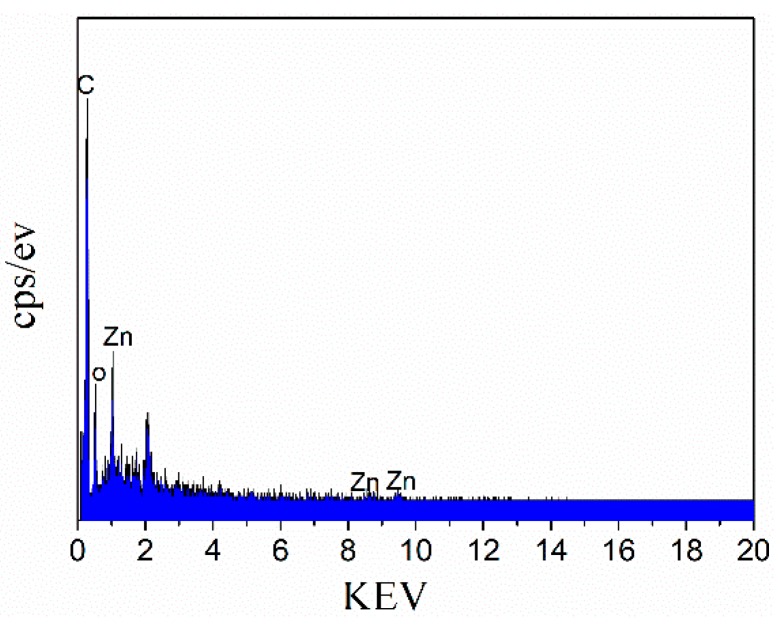
EDS analyses of 4 wt% ZnO NPs-filled fibers.

To verify the materials of PP fibers, EDS was used to determine the chemical composition of the prepared fibers. Figure 4 presents the EDS of 4 wt% ZnO-filled PP. In addition to C and O, Zn can also be observed, indicating the presence of ZnO on the surface of PP fibers.

### 3.4. Differential Scanning Calorimetry Results

**Figure 5:**
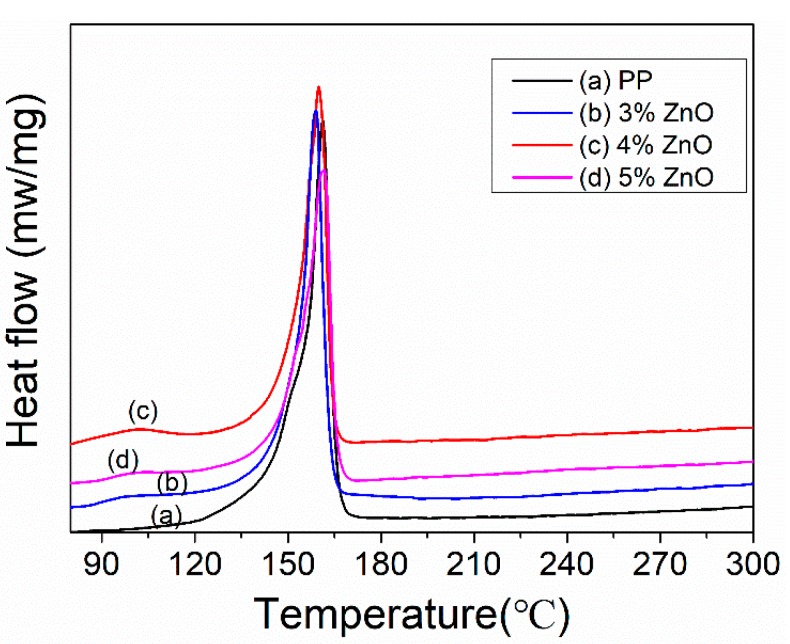
DSC thermograms of (**a**) pure PP (**b**) 3% ZnO-filled fiber (**c**) 4% ZnO-filled fiber (**d**) 5% ZnO-filled fiber.

DSC measurement was performed to investigate crystallization and melting behaviors of ZnO NPs-filled fibers. DSC thermograms and melting temperatures of PP and ZnO NPs-filled fibers are shown in Figure 5 and Table 1, respectively.

Crystallinity of PP fiber grows with the increasing content of ZnO NPs and reaches the maximum at 4 wt%. The crystallinity of pure fiber, 3 wt% ZnO NPs-filled fiber and 4 wt% ZnO NPs-filled fiber are obtained to be 43.75%, 44.46% and 44.62%, respectively. It demonstrates that ZnO NPs can increase the crystallization of PP fiber and function as heterogeneous nucleation [10]. For explanation, ZnO NPs agglomerates will be heterogeneously nucleated after reaching a certain size, thus increasing the crystallization rate of PP. As the content of ZnO NPs increases to 5 wt%, the fiber’s crystallinity reaches 44.06%. It is generally accepted that the aggregates of ZnO NPs are able to hinder crystal growth and thus reduce crystallinity [14].

### 3.5. FTIR Analysis of PP and ZnO NPs Filled PP Fiber with UV Irradiation

**Figure 6:**
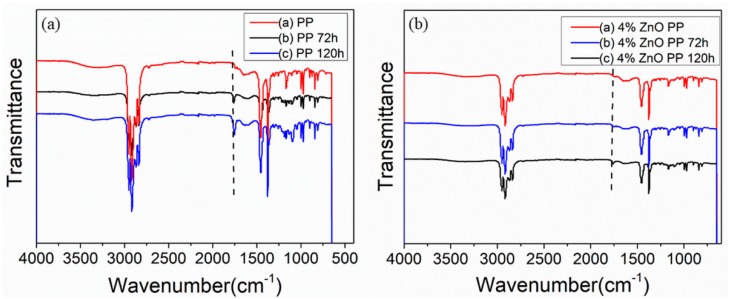
FTIR spectra for (**a**) pure pp (**b**) 4 wt% ZnO NPs-filled pp exposed to UV-light for 0, 72 h and 120 h.

The PP materials are known to be sensitive to the wavelength of over 300 nm, including sunlight and UV light. The photo-degradation of PP materials is invisible and results in the formation of hydroperoxides and carbonyl species. The degradation C=O radical group absorption peaks are found by the FTIR method to be near the wavenumber of 1700 cm^−1^. The photo-degradation degree of PP fiber is closely correlated to the carbonyl content: the smaller the carbonyl content, the low degradation rate the PP fiber. As depicted in Figure 6a, for the pure pp fiber exposed to UV-light for 72 h and 120 h, the absorption peak appears at 1726 cm^−1^. The PP fiber exposed to 120 h of UV-light presents a stronger peak than the fiber exposed to 72h of UV-light. It verifies that the degradation degree of PP fiber has a positive relationship with the time of UV-light irradiation. For the fiber filled with ZnO NPs, as shown in Figure 6b, the absorption peak at 1726 cm^−1^ has no obvious change, confirming that ZnO NPs can inhibit the UV-light degradation of PP fibers.

### 3.6. Antibacterial Activity of ZnO NPs-Filled PP Fiber

The antimicrobial activity of ZnO NPs-filled fibers was evaluated by calculation of the percentage reduction in *E.coli* and *S. aureus* colonies. With pure PP fiber as control, the results are listed in Table 2. Pure PP samples show no antibacterial properties against both *E.coli* and *S. aureus*. By contrast, ZnO NPs-filled PP fibers exhibit good antibacterial activities. Specifically, when the contents of ZnO NPs increase 3% and more, the bacterial reduction rates of both *S. aureus* and *E. coli* reach above 99%.

**Scheme 3:**
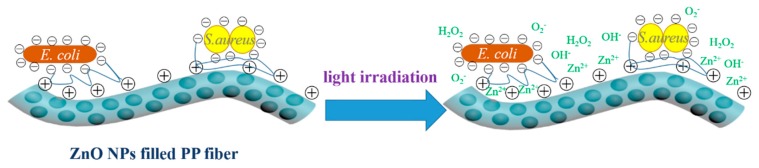
Antimicrobial mechanism ZnO NPs filled fiber.

Zhang et al. reported that a PLA film containing 3% ZnO NPs has antibacterial activity for *S. aureus* and *E. coli* and the inhibition rate was 60% and 58%, respectively. [31] Silvestre et al. reported that PP composites containing 5% ZnO NPs has 85.3 percent reduction of *E. coli* with 24 h. [17] However, in our study, the 3% ZnO NPs filled PP fiber showed better bactericidal efficacy against S. aureus and E.coli, the inhibition rate more than 99%.The antimicrobial mechanism of ZnO NPs-filled PP fiber can be explained from two aspects seen in Scheme 3. Generally, bacteria are negatively charged, which can be adsorbed by positively charged amino HBP via electrostatic adhesion. The direct interaction of HBP-ZnO NPs with bacteria will result in cell inactivation by disrupting the integrity of the cell membrane. In addition, under light irradiation, ZnO NPs on the surface of PP fibers generate reactive oxygen species, contributing to the decomposition and damage of bacterial cells.

## 4. Conclusions

In this paper, the amino capped ZnO NPs were obtained by simply using amino HBP and zinc acetate. ZnO NPs-filled PP fibers were prepared by melt spinning. Moreover, the morphology, thermal property, UV-light degradation behavior, antibacterial activity and mechanical properties of ZnO NPs-filled fibers were determined. The results show the improvement in the mechanical properties, resistance to UV-light degradation and antibacterial activity of ZnO NPs-filled fiber. In particular, the PP fiber with 4 wt% ZnO NPs is proved to be the optimum selection for PP modification.

## Figures and Tables

**Table 1:** Thermal properties of the samples obtained from DSC.

Sample	Melting Temperature (Tm) (°C)	Melting Enthalpy (ΔHm) (Jg^−1^)	Crystalline Ratio (%)
PP	162.16	90.14	43.75
PP/ZnO 3%	162.78	92.05	44.46
PP/ZnO 4%	162.25	92.38	44.62
PP/ZnO 5%	162.18	91.22	44.06

**Table 2:** Antibacterial activity of ZnO NPs-filled PP fiber.

Sample	*S. aureus*		*E. coli*	
	Surviving Cells (CFU/mL)	Reduction (%)	Surviving Cells (CFU/mL)	Reduction (%)
Pure fiber	1.47 × 10^6^	-	2.3 × 10^5^	-
1% ZnO filled fiber	7.5 × 10^5^	64.6	1.2 × 10^5^	47.85
2% ZnO filled fiber	1.1 × 10^5^	92.51	2.6 × 10^4^	88.7
3% ZnO filled fiber	2.4 × 10^3^	99.83	1.6 × 10^3^	99.3
4% ZnO filled fiber	7.2 × 10^2^	99.9	6.5 × 10^2^	99.71
